# Investigating the Synergistic Bactericidal Effects of Cold Plasma and Ultraviolet Radiation on *Pseudomonas fragi*

**DOI:** 10.3390/foods14040550

**Published:** 2025-02-07

**Authors:** Haidu Yuan, Fei Chen, Jiajia Zhang, Xinglei Guo, Jianhao Zhang, Wenjing Yan

**Affiliations:** 1 National Center of Meat Quality & Safety Control, College of Food Science and Technology, Nanjing Agricultural University, Nanjing 210095, China; 2022108072@stu.njau.edu.cn (H.Y.); 2023808193@stu.njau.edu.cn (J.Z.); guoxinglei@stu.njau.edu.cn (X.G.); nau_zjh@njau.edu.cn (J.Z.); 2 Springsnow Food Group Co., Ltd., Yantai 265200, China; lycxcf@126.com

**Keywords:** *Pseudomonas fragi*, cold plasma, ultraviolet, bactericidal effect

## Abstract

Cold plasma is a novel non-thermal processing technology with broad application prospects in food preservation. When combined with other physical sterilization technologies, it enhances sterilization efficiency and broadens its application scope, providing a safe and effective alternative to traditional sterilization methods. In this paper, the sterilization effect of surface dielectric barrier discharge (SDBD) plasma combined with 222 nm ultraviolet (UV) irradiation against *Pseudomonas fragi* (*P. fragi*) was explored for the first time. The sterilization process parameters of SDBD + UV were optimized using the response surface methodology. And the sterilization mechanism of SDBD + UV was preliminary elucidated. The results indicated that the SDBD + UV treatment was highly effective against *P. fragi*. It could eliminate 6.35 Log CFU/g of *P. fragi* within 150 s, establishing optimal sterilization parameters: a radiation distance of 16.4 cm and a saving time (a period of preservation in which the samples were retained in the device after the treatment) of 120 s. Furthermore, the treatment caused significant damage to the cell membrane of *P. fragi*, leading to membrane perforation and content leakage. It also induced oxidative stress, as evidenced by membrane lipid peroxidation, alterations in intracellular reactive oxygen species (ROS) content, and a decrease in antioxidant enzyme activity. This study provides a theoretical basis for the application of cold plasma combined with 222 nm UV treatment in the meat industry.

## 1. Introduction

*Pseudomonas fragi* (*P. fragi*) is a notorious pathogen in the domain of meat and meat product spoilage. This microorganism is notorious for its capacity to proliferate rapidly in the high-moisture, nutrient-rich environment of fresh meat, where it can swiftly outcompete other bacteria and predominate in the microbial flora [1]. The spoilage process instigated by *P. fragi* is characterized not only by the generation of unpleasant odors as a result of the metabolism of amino acids into amines, esters, and sulfides but also by the modification of the meat’s sensory attributes and nutritional constitution [2]. The rapid growth rate and metabolic adaptability of this bacterium make it a formidable adversary in the preservation of meat products, as it can significantly reduce their shelf life and pose health risks if the product is consumed after spoilage has occurred. During metabolism, *P. fragi* may produce harmful substances such as biogenic amines and toxins. Biogenic amines can cause allergic reactions and other discomforts, while toxins can lead to food poisoning [3]. Thus, how to control the growth of *P. fragi* represents a significant importance for improving the quality and extending the shelf life of meat and meat products.

Non-thermal sterilization technologies have received significant attention in recent years due to their ability to inactivate pathogenic microorganisms without the need for heat energy consumption or chemical residues, thereby minimally impacting food quality and extending shelf life [4]. Among these technologies, cold plasma (CP), which can ionize air to produce evenly distributed active bactericidal substances, such as reactive oxygen species (ROS), reactive nitrogen species (RNS), ozone, ultraviolet photons, and charged particles [5], stands out due to its unique advantages, including energy efficiency, sustainable processing, and versatility in sterilizing a variety of materials and shapes. These qualities endow it with the potential to be applied in material technology, food processing, and biomedicine. In the field of food processing, CP is particularly advantageous for its universal sterilization and preservation effects on various solid and liquid foods [6]. CP can be generated using different generators, each with its own set of characteristics and operational principles, such as glow discharge, radio frequency (RF) plasma, and microwave plasma. One commonly used type is a surface dielectric barrier discharge (SDBD) generator. In SDBD, one electrode is covered with a dielectric layer, while the other electrode is typically bare. The dielectric layer serves to distribute the electric field uniformly and prevent the formation of arc discharges. When a high voltage is applied, the electric field at the surface of the dielectric layer becomes very strong, leading to the ionization of the gas in contact with the surface. This ionization produces a thin layer of plasma, which can be controlled by adjusting the applied voltage and the properties of the dielectric material. SDBD generators are known for their ability to create cold plasma with high diffusivity and a large surface area. This makes them particularly suitable for sterilization in food products with irregular shapes and sizes [7,8,9,10]. In one study, an SDBD treatment (3 kV, 45 kHz, applied for 15 min at 25 °C) effectively reduced the initial total microbial load of fresh sea bream fillets by 1.03 Log CFU/g and also diminished fish odor, as reported by Giannoglou [11]. However, the SDBD sterilization technology alone has limitations. It exhibits a suboptimal killing effect on certain microorganisms and may struggle with adapting to the specific structures of certain foods. Achieving uniformity in SDBD can be challenging due to variations in dielectric layer thickness, surface roughness, and electrode geometry, leading to non-uniform discharge patterns and edge effects. Additionally, SDBD requires high voltages, resulting in higher energy consumption and increased operational costs.

The integration of plasma with other non-thermal sterilization technologies is an important research trend in food processing. It helps to improve sterilization efficiency, ensure food safety, and minimize the impact on the nutritional and sensory quality of food. One report indicated that the combination use of SDBD and H_2_O_2_ could reduce the number of *P. tolaasii* strain Pt18 on the mushroom surface by approximately 2.5 Log CFU/filter after 180 s of treatment, which was significantly higher than that of SDBD treatment [12]. Ultrasonic-assisted DBD plasma technology, as demonstrated by Pan et al. [13], effectively sterilized *Listeria monocytogenes* across a range of temperatures (10, 25, 37 and 42 °C). Temperature and ultrasonic-assisted plasma significantly improve the sterilization rate by affecting bacterial cell membranes and reducing their resistance to plasma. UV sterilization is another effective non-thermal processing technology that primarily works by damaging the DNA of microorganisms, forming pyrimidine dimers that disrupt replication, and by denaturing proteins, which affects cellular structure and function. UV also generates ROS that cause oxidative stress, leading to cell death [14]. Among UV technologies, 222 nm far-ultraviolet excimer lamps demonstrate their potential use in the food industry due to their environmental friendliness, extended service life, and the low irradiation doses they require [15,16]. However, UV’s effectiveness is limited by its surface treatment nature and shadowing effects, where objects or packaging can block the light, reducing its impact. Currently, research on the combined treatment of SDBD plasma and UV is primarily focused on the degradation of harmful gases, toxins, and antibiotics, with limited studies on the sanitization of harmful microorganisms in food [17].

In this study, the bactericidal effect and underlying mechanism of the combined treatment of SDBD plasma and 222 nm UV radiation on *P. fragi* were investigated. Firstly, a comparison is made regarding the differences in the bactericidal efficacy of SDBD, UV, and SDBD + UV treatments against *P. fragi*. The response surface method was employed to explore the optimal sterilization process parameters for the SDBD + UV treatment when dealing with *P. fragi*. The optical emission spectroscopy (OES) of the single-factor treatment or combination treatment was determined to understand its optical characteristics. The bactericidal mechanism is delved into from multiple aspects, including morphological observation, non-viable bacteria staining, content leakage assessment, and oxidative stress analysis. This introduction lays the groundwork for a comprehensive exploration of the potential of this combined treatment approach in sterilization and preservation applications.

## 2. Materials and Methods

### 2.1. Materials

*P. fragi* (BNCC 134017^TM^) was obtained from BeNa Culture Collection, Beijing, China. A propidium iodide staining solution was sourced from Nantong FeiYu Biotechnology Co., Ltd., Nantong, China, while a Bicinchoninic Acid Assay (BCA) Protein Content Assay Kit and 2′,7′-Dichlorodihydrofluorescein diacetate (DCFH-DA) fluorescent probe were acquired from Shanghai Bestbio Biotechnology Co., Ltd., Shanghai, China, and Beijing Solarbio Science & Technology Co., Ltd., Beijing, China, respectively.

### 2.2. Bacterial Culture and Treatment

*P. fragi* strains were incubated in Tryptone Soy Broth (TSB) at 30 °C for 7 h to reach the logarithmic growth phase. The bacterial suspension was then centrifuged at 6800 rpm for 8 min at 4 °C, after which the supernatant was removed, and the pellet was washed twice with 0.01 M phosphate-buffered saline (PBS, pH 7.2). The *P. fragi* concentration was adjusted to 8.4 ± 0.2 Log CFU/g, as determined using a Shimadzu 2600 UV-vis spectrophotometer from Shimadzu Corporation, Kyoto, Japan at 600 nm, which is based on the principle called the McFarland turbidity method that bacterial concentration is directly proportional to turbidity. When the OD600 is 0.7, the concentration of *P. fragi* is approximately 8.4 Log CFU/g.

**Figure 1:**
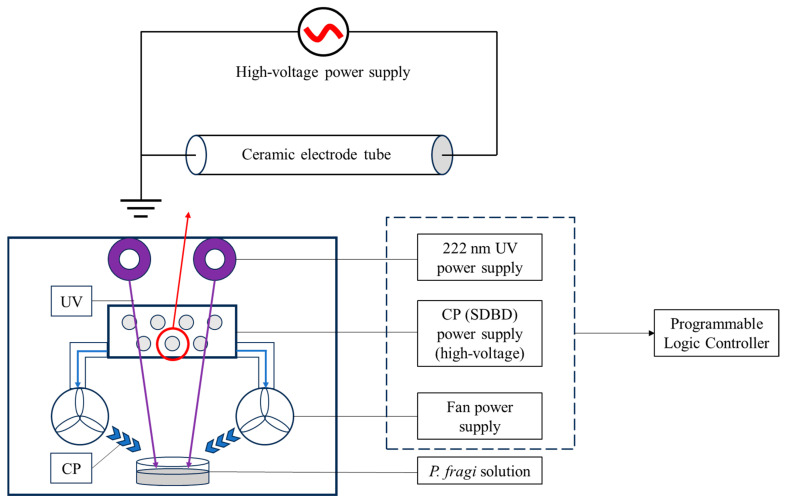
Schematic diagram of the SDBD + UV treatment device.

An amount of 20 mL of the *P. fragi* suspension was added to the plate and then put into the SDBD device with 222 nm excimer lamps and fans, and the bacterial cells were treated under different conditions. Then, the solutions were collected, and the 10-fold serially diluted cells were spotted onto plate count agar (PCA). The CFUs were counted after incubation at 30 °C for 24 h, and the results were expressed as Log colony-forming units per gram (Log CFU/g). During the treatment, UV rays are emitted vertically from the top of the device, while the plasma-active materials produced by the SDBD element are blown horizontally from fans underneath the device. After the end of the treatment, the sample is allowed to undergo a period of preservation in the device, and finally, the remaining plasma-active materials are expelled from the device by fans (Figure 1).

### 2.3. Optimization of Sterilization Process of SDBD + UV

#### 2.3.1. Single-Factor Experiment

As shown in Table 1, the effects of different treatment times (30, 60, 90, 120, and 150 s) on the sterilization effect were studied under the conditions of a fixed irradiation distance (18 cm) and saving time (90 s). The effects of different irradiation distances (6, 12, 18, 24, and 30 cm) on the sterilization effect were studied under the conditions of a fixed treatment time (90 s) and saving time (90 s). The effects of different saving times (30, 60, 90, 120, and 150 s) on the sterilization effect were studied under the conditions of the fixed treatment time (90 s) and irradiation distance (18 cm).

#### 2.3.2. Response Surface Methodology (RSM)

Based on the results of the single-factor test, a three-factor and three-level Box–Behnken test was designed by the Design-Expert V8.0.6 software, in which treatment time, irradiation distance, and saving time were denoted as factors A, B, and C, respectively. The reduction in *P. fragi* served as the response variable, allowing for a comprehensive understanding of the disinfection dynamics of the SDBD + UV treatment and obtaining the optimal sterilization conditions.

### 2.4. Optical Emission Spectroscopy (OES) Qualitative Testing

The optical emission spectroscopy (OES) was conducted using a high-resolution spectrometer (HR +2000, Ocean Optics, Inc., Orlando, FL, USA) with the container of the SDBD/222 nm UV-integrated sterilization device. A fiber spectrometer was employed to measure the characteristics of the active substances produced during the SDBD + UV treatment process. The spectral range was 200–1050 nm. An optical fiber with a 1000 μm core diameter and 0.22 numerical aperture transmitted the plasma emission light source. The fiber optic probe was fixed midway in the container to capture the optical signal. All the components mentioned are from Ocean Optics. The spectral data were analyzed using the atomic spectroscopy database and methods from the American Institute of Standards and Technology. The qualitative analysis of plasma-active species was determined based on the position and shape of the signal peaks in the spectral diagram [17,18].

### 2.5. Scanning Electron Microscopy (SEM) Analysis

For the SEM observation, bacterial suspensions (10^8^ CFU/mL) after treatment with UV, SDBD, and SDBD + UV, respectively, were harvested via centrifugation (3000 rpm, 10 min) at 4 °C, and then the bacteria were fixed by 2.5% glutaraldehyde and rinsed with sterile PBS. The solution was dehydrated with ethanol systematically then freeze-dried by vacuum and sputter gold-plated before observation by SEM at 20,000× magnification (Regulus 8100, Hitachi, Tokyo, Japan).

### 2.6. Plasma Membrane Integrity

#### 2.6.1. PI Staining

Bacterial suspensions (10^8^ CFU/mL) treated with UV, SDBD, and SDBD + UV, respectively, were harvested and resuspended in sterile PBS. An amount of 1 mL of each suspension was stained with 100 μL of propidium iodide (PI) dye (100 μg/mL) at 4 °C for 30 min. The samples were then centrifuged and washed three times with sterile PBS. A 10 μL drop of each sample was applied to a glass slide, covered with a coverslip, and examined under confocal laser scanning microscopy (CLSM, Olympus FV3000, from Olympus Corporation, Tokyo, Japan) at 400× magnification. The excitation wavelength was 488 nm, and the emission wavelength was 549 nm.

#### 2.6.2. Determination of Bacterial Protein Content

The bacterial suspensions were treated under the optimal conditions for 0–150 s followed by centrifugation at 6800 rpm for 8 min. The pellets were washed in PBS three times and ultrasonicated (80% power, 3 s pulses, 3 s intervals, for 8 min) until the solution cleared. The supernatant was retained after centrifugation at 4 °C and 6800 rpm for 8 min, and the total protein content was determined using the BCA kit (Thermo Fisher Scientific, Waltham, MA, USA).

#### 2.6.3. Determination of Protein and Nucleic Acid Leakage

The bacterial suspensions were treated under the optimal conditions for 0–150 s followed by centrifugation at 6800 rpm for 8 min. The supernatant was harvested and used for the determination of protein and nucleic acid leakage of the bacteria. The protein leakage was determined according to the instructions of the BCA kit. The nucleic acid leakage was determined using the Shimadzu 2600 UV-vis spectrophotometer by measuring the absorbance value at a wavelength of 260 nm [19].

#### 2.6.4. Determination of Potassium Ion Leakage

The bacterial suspensions were treated under the optimal conditions for 0–150 s followed by centrifugation at 6800 rpm for 8 min. The supernatant was harvested, and the potassium ion content was determined via a potassium content detection kit (100T, Nanjing Jiancheng Bioengineering Institute, Nanjing, China).

### 2.7. Determination of Intracellular Reactive Oxygen Species

The intracellular reactive oxygen species of bacteria after treatment was determined according to the previous report [20]. The bacterial solutions after treatment were collected and centrifuged at 5000 rpm for 5 min at 4 °C. The plaques were washed 3 times with PBS and resuspended in 0.1 M PBS. An equal volume of 20 μM fluorescent dye DCFH-DA was added, and the mixture was incubated at 30 °C for 30 min in the dark mixed with low-speed shaking. The cells were then washed in 0.1 M PBS and resuspended in PBS at 30 °C for 15 min, and their fluorescence intensity at 485/528 nm (excitation/emission) was determined using a microplate reader, from Thermo Fisher Scientific, Waltham, MA, USA.

### 2.8. Determination of Lipid Peroxidation of Cell Membranes

The thiobarbituric acid (TBA) method was used to evaluate the lipid oxidation of bacterial membranes [21]. Bacterial pellets from each treatment group were treated with 3 mL of a 7.5% trichloroacetic acid solution and disrupted using a disruptor (80% power, 3 s sonication, 3 s intervals, for 8 min). After centrifugation at 4 °C (10,000 rpm for 10 min), the supernatant was aspirated, and 3 mL of a 0.02 M TBA solution was added, vortexed, and heated in a water bath at 90 °C for 30 min. The mixture was cooled on ice and centrifuged at 25 °C (12,000 rpm for 10 min), and the supernatant was used to measure absorbance values at 532 and 600 nm. Malondialdehyde (MDA) content was calculated in terms of bacterial wet weight as nmol/g sample. The formula is as follows:

(1) $$\mathrm{MDA}\;\mathrm{content}=\frac{c\times V}{m}$$

In the formula, *c* is the concentration of malondialdehyde in the sample solution obtained from the standard calibration curve, with units of nmol/mL. *V* is the final volume of the sample solution, with units of mL. And *m* is the wet weight of the bacteria, with units of g.

### 2.9. Determination of Superoxide Dismutase (SOD) and Catalase (CAT) Activity

The SOD and CAT activity of the bacteria after treatment was determined according to the previous report [22]. After treatment for 0–150 s, the bacterial solutions were centrifuged at 6800 rpm for 8 min. An amount of 10 mL of sterile PBS was added to the plaque and sonicated on ice for disruption (80% power, 3 s pulses, 3 s intervals, for 8 min), and then centrifugation was performed to obtain the supernatant. The collected supernatants were utilized for an enzyme activity assay via a Superoxide Dismutase Assay Kit with WST-8 (Beyotime Biotech, Shanghai, China) and a Catalase (CAT) Assay Kit (Nanjing Jiancheng Bio. Inst., Nanjing, China), respectively.

### 2.10. Statistical Analysis

Experiments were conducted in triplicate and analyzed using IBM SPSS Statistics 20.0 software with one-way ANOVA. Duncan’s multiple comparison test (*p* < 0.05) was applied for significance analysis, and data were graphed using Origin 2024 (10.5.128) software.

## 3. Results and Discussion

### 3.1. Single-Factor Test

**Figure 2:**
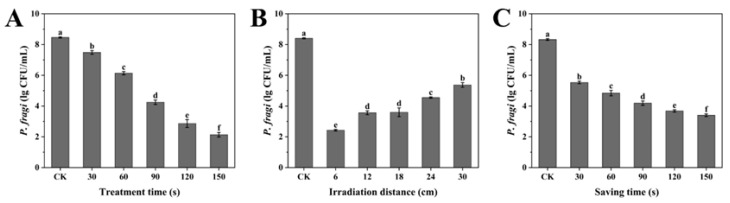
Single-factor experiment effects of three independent variables on the antibacterial activity of SDBD + UV: (**A**) treatment time (irradiation distance: 18 cm, saving time: 90 s); (**B**) irradiation distance (treatment time: 90 s, saving time: 90 s); (**C**) saving time (treatment time: 90 s, irradiation distance: 18 cm). Different letters in the figure indicate significant differences between the data (*p* < 0.05).

As depicted in Figure 2A, the reduction in the number of *P. fragi* was significantly increased with the increasing duration of the SDBD + UV treatment within the 30–150 s range (*p* < 0.05), suggesting a time-dependent bactericidal effect. Figure 2B illustrates the effect of irradiation distance on the sterilization efficacy. The number of *P. fragi* increased as the irradiation distance increased from 6 to 30 cm. However, no statistically significant changes were observed in the 12–18 cm range, which might be related to the structure of the device (Figure 1). The active substances generated by SDBD were dispersed via the air supply system underneath the device, while the UV irradiation was emitted from the excimer lamp above it. As the irradiation distance grew, the sample became farther from the excimer lamp and nearer to the air supply system, implying that there could be an optimal irradiation distance for sterilization within the 12–18 cm range. Considering that the surface temperature increases more significantly with longer treatment times and shorter irradiation distances, to minimize the impact of temperature changes on food quality, a treatment time of 90 s and an irradiation distance of 18 cm were selected. Both the trends are consistent with previous studies, demonstrating the effects of SDBD + UV treatment time and irradiation distance on sterilization efficacy [23]. As shown in Figure 2C, the bacterial count dropped sharply between 30 and 90 s, then slowed. Because the plasma-active bactericidal substances dissipated with the extension of saving time, their concentration became small after 90 s. Based on the above results, 90 s, 18 cm, and 90 s were selected as the design midpoint of treatment time, irradiation distance, and saving time in the response surface optimization test.

### 3.2. Response Surface Optimization Test

Response surface model optimization offers several advantages over traditional single-variable optimization, including saving time, space, and raw materials [24]. Table 2 shows the results of the response surface optimization experiments designed using the Box–Behnken method. The model is extremely significant (*p* < 0.0001) following the data processing and analysis, suggesting that the multiple regression equation accurately fits the experimental data and is statistically significant. As shown in Table 3, the non-significant lack of fit (*p* = 0.7818 > 0.05) indicates a good model alignment with the actual test results. The determination coefficient R^2^ = 0.9942 and the adjusted determination coefficient R^2^ = 0.9867 further confirm the model’s ability to effectively explain and predict the impact of various factors on the response value. The variance analysis revealed that the experimental model significantly affected the response values (*p* < 0.01), with the square term A^2^ significantly influencing the response (*p* < 0.05), while the interaction terms AB, AC and the square term C^2^ had no significant effect (*p* > 0.05). Based on the *F*-values, the influence of the three factors on the response values was ranked as A (treatment time) > B (irradiation distance) > C (saving time).

Through a multiple regression analysis of the experimental data, a mathematical model was derived to represent the reduction in *P. fragi* as a function of the independent variable within the study area, as follows:

R = −2.9650 + 0.08996A + 0.05000B + 4.1667 × 10^−5^C + 9.7222×10^−5^AB + 4.4444 × 10^−5^AC + 2.0139 × 10^−3^BC − 2.4167 × 10^−4^A^2^ − 9.1667 × 10^−3^B^2^ − 9.7222 × 10^−5^C^2^

**Figure 3:**
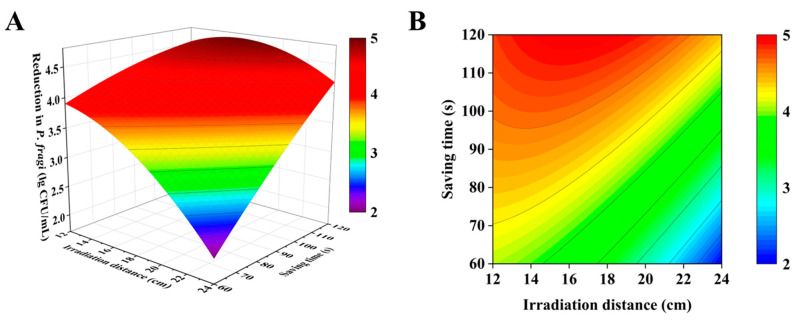
(**A**) Response surface and (**B**) contour plots showing the interactive effects of irradiation distance and saving time on the reduction in *P. fragi* (the fixed treatment time: 90 s).

Based on this formula, a significant interaction irradiation distance (B) and saving time (C) was observed, as depicted by the response surface and contour plots (Figure 3). The sterilization effect was negatively correlated with the irradiation distance when the saving time was short, aligning with the inverse relationship between UV irradiation distance and energy density. However, for longer saving times, a greater irradiation distance was needed for optimal sterilization. This could be due to the plasma-active bactericidal substances being produced in the lower layer of the device during the treatment, leading to a higher concentration in the lower layer. With extended saving times, these substances are more likely to contact the sample in the lower space, where a longer irradiation distance is required for better bactericidal effects. The formula determined that for maximum *P. fragi* reduction with the SDBD + UV treatment, the optimal settings are an irradiation distance of 16.4 cm and a saving time of 120 s, which were then used to study the bactericidal effects and mechanism of *P. fragi* at various treatment times.

### 3.3. Sterilization Test of SDBD + UV Treatment

**Figure 4:**
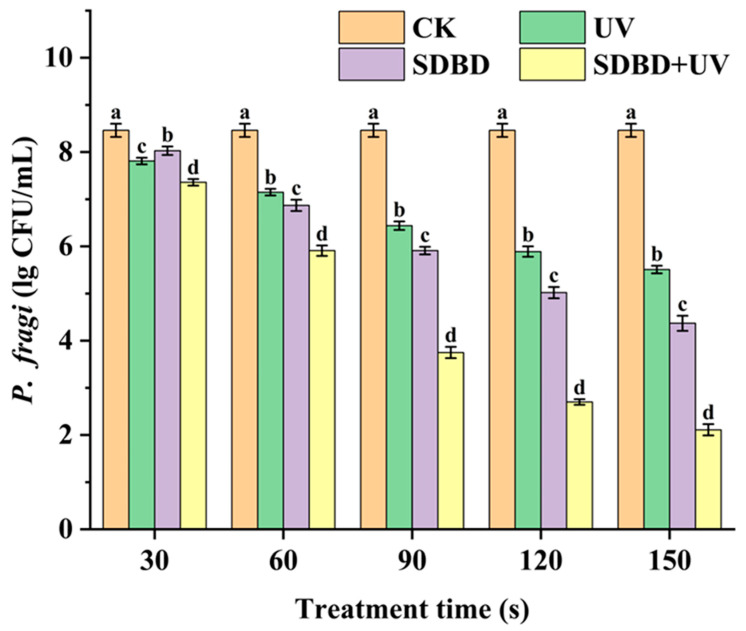
The bactericidal effects of SDBD, UV, and SDBD + UV treatments on *P. fragi*. Different letters in the figure indicate significant differences between the data (*p* < 0.05).

As shown in Figure 4, the combined SDBD with UV treatment demonstrates a markedly stronger bactericidal effect on *P. fragi* compared to either treatment alone, consistent with previous research [23]. At 30 s of treatment, the UV group exhibited a 0.65 Log CFU/g reduction in *P. fragi*, slightly higher than the SDBD group’s 0.43 Log CFU/g reduction. The SDBD + UV group showed the most significant decrease at 1.10 Log CFU/g. Extending the treatment to 60 s, the UV group’s reduction was 1.31 Log CFU/g lower than the SDBD group’s 1.59 Log CFU/g, while the SDBD + UV group had the largest reduction at 2.55 Log CFU/g. The SDBD treatment was more effective than UV alone, but the SDBD + UV treatment was consistently the most lethal. This could be attributed to the initial low level of plasma-active materials like ozone and nitrogen oxides, which increase with treatment time, becoming effective at killing microorganisms. At 150 s, the reductions in *P. fragi* counts for the UV, SDBD, and SDBD + UV groups were 2.95, 4.09, and 6.35 Log CFU/g, respectively.

### 3.4. OES Qualitative Testing

**Figure 5:**
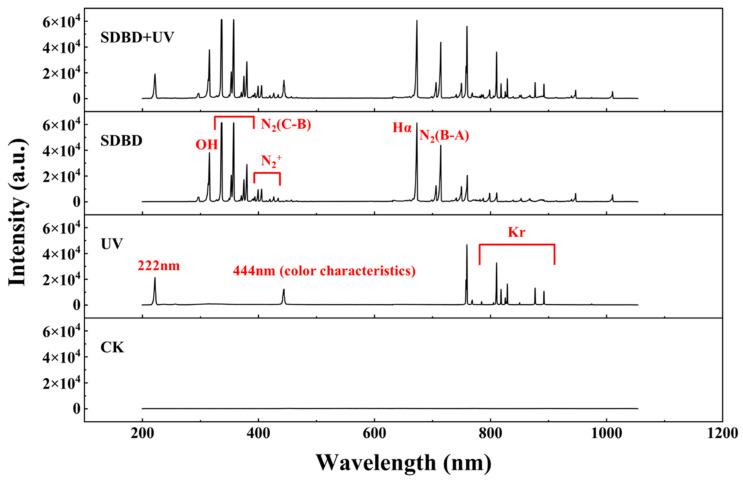
The OES diagram of the SDBD, UV, and SDBD + UV treatments, ranging from 200 to 1150 nm.

OES is a powerful diagnostic tool used to analyze the active species generated during the operation of SDBD and UV treatments. SDBD generates a variety of active species, including ROS, reactive nitrogen species (RNS), and charged particles. These species emit light at specific wavelengths when they transition from excited states to lower energy levels. UV radiation, particularly in the 200–300 nm range, can cause ionization and excitation of gas molecules, leading to the emission of characteristic spectral lines [25,26]. Figure 5 shows the OES diagram of the SDBD, UV, and SDBD + UV treatments between 200 and 1150 nm. It can be seen that the emission spectrum of the SDBD is primarily dominated by nitrogen emissions (N_2_ (C–B): 300–420 nm, N_2_^+^: 400–520 nm, N_2_ (B–A): 300–420 nm), OH emissions at 310 nm, and Hα emissions at 672 nm. The UV emission peaks are mainly characterized by Kr lines at 810, 819, 829, 877, and 886 nm, as well as a peak at 222 nm. The emission spectrum of the SDBD + UV treatment exhibits all the characteristic peaks of both SDBD and UV, indicating that the physical processes of SDBD and UV can be effectively integrated.

### 3.5. Observation of Cell Structure

**Figure 6:**
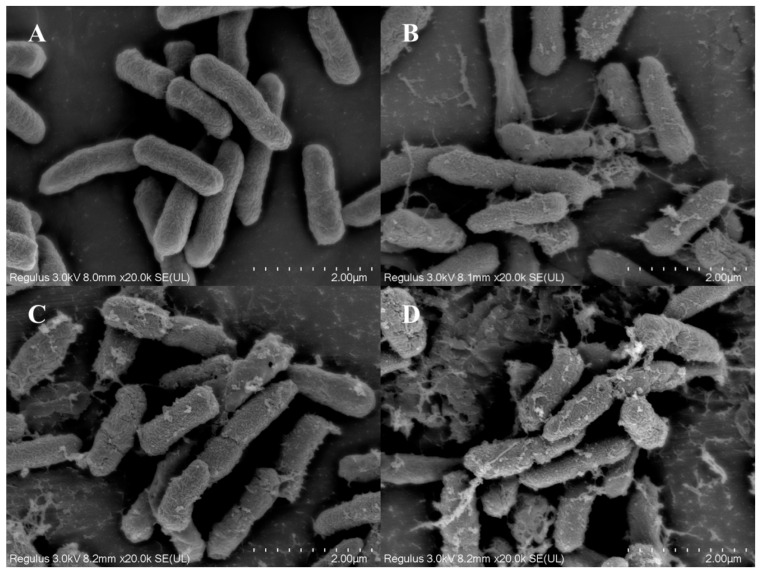
SEM images of *P. fragi* after different treatments: (**A**) control group; (**B**) UV treatment; (**C**) SDBD treatment; (**D**) SDBD + UV treatment.

SEM is a powerful tool for observing the morphological changes in bacterial cells. Untreated bacterial cells typically appear intact with smooth cell membranes and no signs of damage. The cells maintain their natural shape and structure, which is essential for their viability and function [27]. Figure 6 presents the SEM images of *P. fragi* cells after treatment with UV, SDBD, and SDBD + UV for 2 min, respectively. It was observed that bacterial cells in the untreated control group remained intact, with no signs of cell membrane shrinkage or perforation. In contrast, the cell membrane of the bacteria treated with UV and SDBD showed signs of shrinkage, with the appearance of holes and a minor leakage of cellular contents. The most serious damage was observed in the SDBD + UV treatment group. The cell membranes showed extensive shrinkage, larger holes, and significant content leakage. And cell disintegration occurred, indicating a more profound impact on the cell structure. The bacterial surfaces were extensively damaged, leading to substantial leakage of cellular contents and even some instances of cell disintegration.

### 3.6. PI Staining

**Figure 7:**
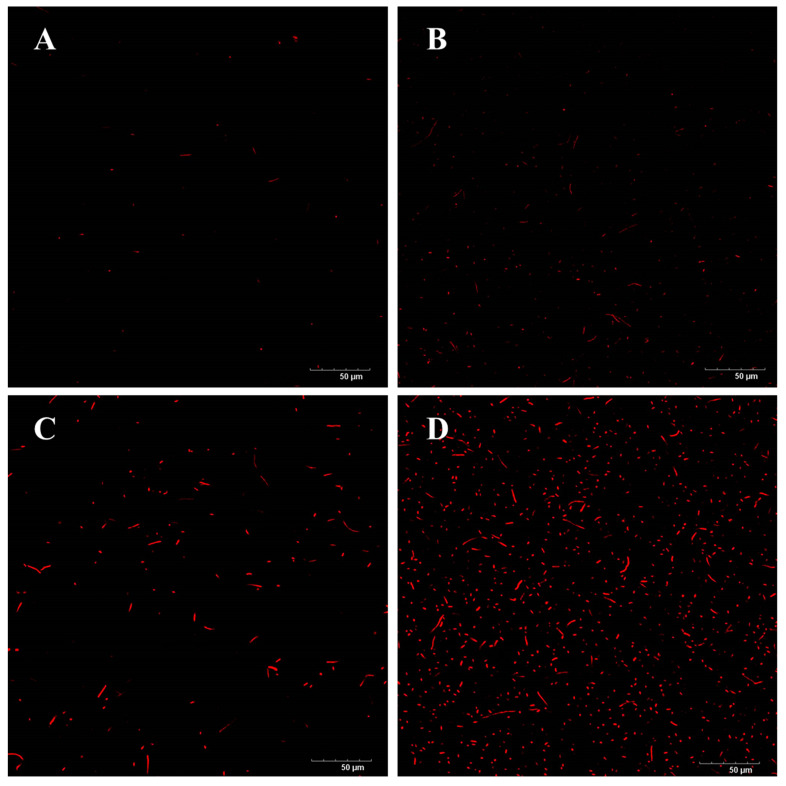
CLSM images of *P. fragi* after different treatments followed by staining with PI dye: (**A**) control group; (**B**) UV treatment; (**C**) SDBD treatment; (**D**) SDBD + UV treatment.

The impact of the SDBD + UV treatment on the membrane permeability of *P. fragi* was further analyzed using a combination of PI fluorescence staining and confocal laser scanning microscopy (CLSM). PI is a nucleic acid dye that binds to DNA and emits red fluorescence only after the cell membrane has been compromised [28]. As shown in Figure 7A, the CLSM image of the control group exhibits minimal red fluorescence, indicating that the cell membranes of *P. fragi* remain intact. However, Figure 7B demonstrates an increase in red fluorescence after the UV treatment, suggesting that a significant number of bacterial cell membranes have been ruptured. The red fluorescence intensity in Figure 7C is more pronounced, indicating that the SDBD treatment has a more potent damaging effect on bacterial cell membranes compared to the UV treatment. This enhanced effect may be attributed to the accumulation of plasma-active substances in a confined environment, leading to a persistent impact on bacterial cell membranes that surpasses the bactericidal effect of UV radiation. The damaging effect of SDBD + UV treatment on the cell membranes of *P. fragi* is further evidenced in Figure 7D, which displays the highest intensity of red fluorescence, aligning with its most effective bactericidal action.

### 3.7. Determination of Protein Content and Leakage of Bacterial Contents

**Figure 8:**
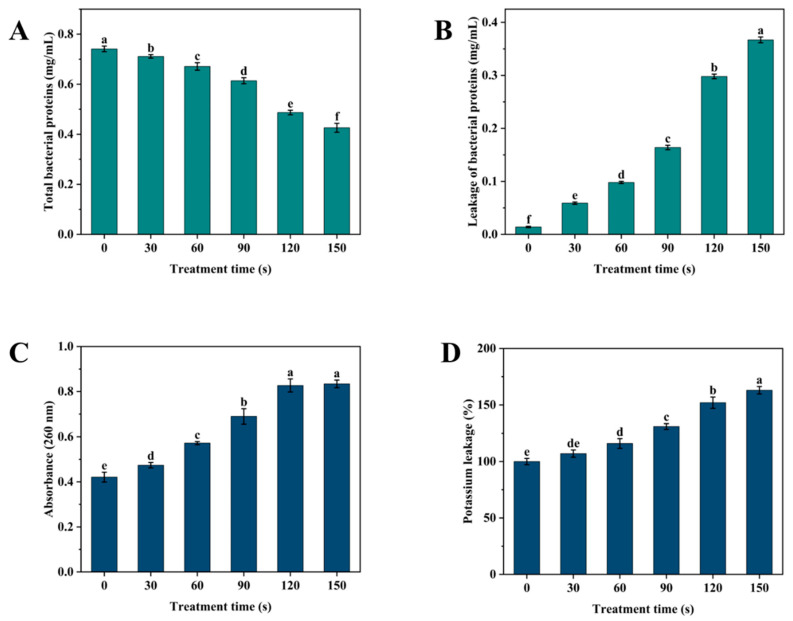
Effects of SDBD + UV treatment on (**A**) protein content, leakage of (**B**) protein, (**C**) nucleic acid, and (**D**) potassium leakage of *P. fragi* over the period of 0–150s. Different letters in the figure indicate significant differences between the data (*p* < 0.05).

The total protein content and leakage from bacterial cells can indirectly reflect the extent of damage to bacterial cell membranes. Figure 8A,B demonstrates that as the SDBD + UV treatment time increases, the total protein content of the bacterial cells decreases. Initially, the decrease is gradual, but it reaches 0.426 mg/mL at 150 s of SDBD + UV treatment time, reducing 0.315 mg/mL of the *P. fragi*. Concurrently, protein leakage increases to 0.367 mg/mL, indicating that the SDBD + UV treatment causes the intracellular proteins of *P. fragi* to leak outside the cell through the damaged membrane. Similarly, Figure 8C,D illustrates the effect of SDBD + UV on the permeability of cell membranes through nucleic acid and potassium ion leakage. With the increase in SDBD + UV treatment time, both the OD_260_ and potassium ion content of the solution exhibit an upward trend. When the treatment time reaches 150 s, the OD_260_ reaches 0.82 and the potassium ion leakage reaches 163% of that in the control group. This phenomenon indicates that the continuous effect of the SDBD + UV treatment leads to a gradual increase in the permeability of bacterial cell membranes, thereby enhancing the leakage of potassium ions [29].

### 3.8. Mechanisms of Oxidative Stress

**Figure 9:**
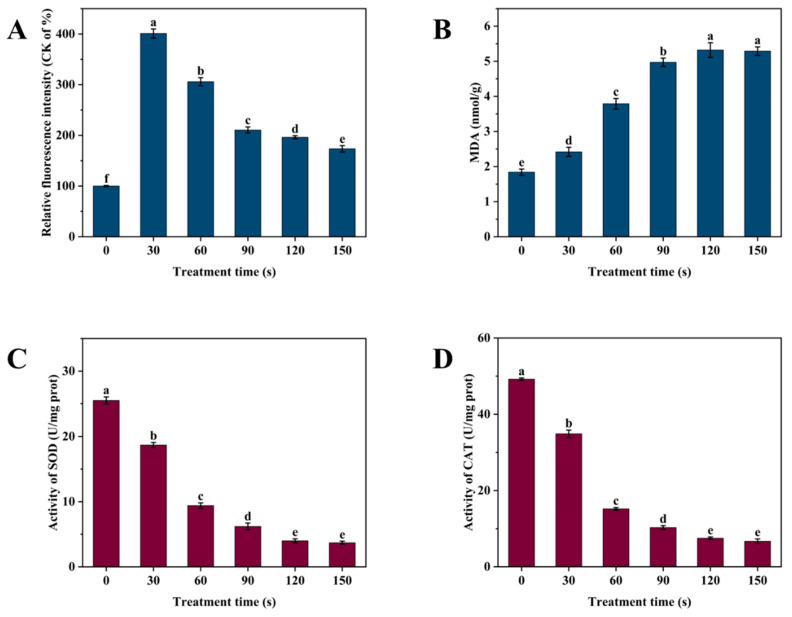
(**A**) ROS content, (**B**) membrane lipid peroxidation, and (**C**) SOD and (**D**) CAT enzyme activity of *P. fragi* after SDBD + UV treatment over the period of 0–150 s. Different letters in the figure indicate significant differences between the data (*p* < 0.05).

Elevated levels of ROS and UV irradiation are known to induce oxidative stress in cells [30,31]. In this study, we assessed the oxidative stress in *P. fragi* induced by SDBD + UV treatment by measuring intracellular ROS levels using a DCFH-DA fluorescent probe under various treatment times. Figure 9A shows that the intracellular ROS in *P. fragi* initially increased and subsequently decreased with the extension of the SDBD + UV treatment time, peaking at 401.0% of the control level at 30 s before gradually decreasing, yet remaining higher than that of the control group. Lipid peroxidation in the cell membrane is a direct indicator of cellular oxidative stress. Figure 9B indicates that membrane lipid peroxidation in *P. fragi* increased with treatment time, reaching a stable level at 120 s with an MDA content of 5.32 nmol/g, significantly higher than the control group’s 1.84 nmol/g.

Excess ROS production typically triggers the action of antioxidant enzymes to maintain bacterial redox balance [32]. Figure 9C,D illustrates that the activities of SOD and CAT enzymes in the control group were 25.5 and 49.2 U/mg protein, respectively. These activities decreased with treatment time, particularly within the first 60 s, dropping to 9.4 and 15.2 U/mg protein, respectively. This suggests that as SDBD + UV treatment time increases, cellular oxidative stress quickly overwhelms the enzyme system’s defense, leading to a significant accumulation of ROS that cannot be effectively neutralized. When ROS levels exceed a threshold, they damage the antioxidant enzyme system, affecting the activities of SOD and CAT [33]. The accumulation of ROS leads to excessive oxidative stress, causing oxygen toxicity in cells and hastening the inactivation of SOD and CAT.

## 4. Conclusions

In conclusion, we investigated the effects of SDBD + UV treatment on *P. fragi* and preliminary revealed the bactericidal schematic. We found that SDBD + UV treatment can significantly reduce *P. fragi* populations, with a reduction of 6.35 Log CFU/g for the SDBD + UV treatment lasting 150 s under the optimal parameters (radiation distance: 16.4 cm, saving time: 120 s). The bactericidal mechanism involves the destruction of cell structure, increased membrane permeability, and oxidative stress induction, leading to cellular damage and death. This study provides a method and theoretical foundation for the field of sterilization and preservation in the meat industry. While our study provides valuable insights into the efficacy of SDBD + UV treatment against *P. fragi,* it is limited to this specific bacterium. Future research may explore the effects of this treatment on a broader spectrum of microorganisms to establish its universality and applicability. Additionally, studies on the impact of SDBD + UV treatment on meat quality and sensory attributes are warranted to fully understand its practical implications.

## Figures and Tables

**Table 1:** Factors and levels tested for the designed experiment.

Factor	Low Level (−1)	Medium Level (0)	High Level (1)
Treatment time (s)	60	90	120
Irradiation distance (cm)	12	18	24
Saving time (s)	60	90	120

**Table 2:** Box–Behnken design and the response for the reduction in *P. fragi*.

Experiments	Coded Levels			Reduction in *P. Fragi* (lg CFU/mL)
	A	B	C	
	Treatment Time (s)	Irradiation Distance (cm)	Saving Time (s)	
1	60 (−1)	12 (−1)	90 (0)	2.52
2	120 (1)	12 (−1)	90 (0)	5.56
3	60 (−1)	24 (1)	90 (0)	1.51
4	120 (1)	24 (1)	90 (0)	4.62
5	60 (−1)	18 (0)	60 (−1)	1.53
6	120 (1)	18 (0)	60 (−1)	4.64
7	60 (−1)	18 (0)	120 (1)	2.87
8	120 (1)	18 (0)	120 (1)	6.14
9	90 (0)	12 (−1)	60 (−1)	3.98
10	90 (0)	24 (1)	60 (−1)	2.07
11	90 (0)	12 (−1)	120 (1)	4.57
12	90 (0)	24 (1)	120 (1)	4.11
13	90 (0)	18 (0)	90 (0)	4.11
14	90 (0)	18 (0)	90 (0)	4.2
15	90 (0)	18 (0)	90 (0)	4.32
16	90 (0)	18 (0)	90 (0)	3.86
17	90 (0)	18 (0)	90 (0)	4.01

**Table 3:** Analysis of variance and test of significance for the regression coefficient for the response surface quadratic model for the reduction in *P. fragi*.

Source	Sum of Squares	df	Mean Square	*F*-Value	*p*-Value Prob > F
Model	26.98	9	3	132.51	<0.0001
A-A	19.63	1	19.63	867.41	<0.0001
B-B	2.33	1	2.33	103.11	<0.0001
C-C	3.74	1	3.74	165.31	<0.0001
AB	1.225 × 10^−3^	1	1.225 × 10^−3^	0.054	0.8227
AC	6.4 × 10^−3^	1	6.4 × 10^−3^	0.28	0.6113
BC	0.53	1	0.53	23.23	0.0019
A^2^	0.2	1	0.2	8.8	0.0209
B^2^	0.46	1	0.46	20.27	0.0028
C^2^	0.032	1	0.032	1.42	0.2715
Residual	0.16	7	0.023		
Lack of Fit	0.034	3	0.011	0.37	0.7818
Pure Error	0.12	4	0.031		
Cor Total	27.14	16			
	R-squared = 0.9848		Adj R-squared = 0.9652		

## Data Availability

The original contributions presented in the study are included in the article, further inquiries can be directed to the corresponding author.
